# Hemoperitoneum From Bleeding Intra-Abdominal Varices: A Rare, Life-Threatening Cause of Abdominal Pain in a Patient With Cirrhosis

**DOI:** 10.7759/cureus.18955

**Published:** 2021-10-21

**Authors:** Nicha Wongjarupong, Hamdi S Said, Richie K Huynh, Jafar Golzarian, Nicholas Lim

**Affiliations:** 1 Internal Medicine, University of Minnesota, Minneapolis, USA; 2 Division of Gastroenterology, Hepatology, and Nutrition, University of Minnesota, Minneapolis, USA; 3 Gastroenterology and Hepatology, University of Minnesota, Minneapolis, USA; 4 Medicine, M Health Fairview Woodwinds Hospital, Woodbury, USA; 5 Interventional Radiology, University of Minnesota, Minneapolis, USA

**Keywords:** extrahepatic portal hypertension, ir guided embolization, alcoholic cirrhosis, intraperitoneal bleeding, ectopic varices

## Abstract

We report the case of a 54-year-old male with alcoholic cirrhosis who presented several times to the emergency department (ED) with right upper quadrant abdominal pain. Ten days after his initial presentation, the patient represented with hypotension and anemia. An abdominal CT angiogram identified hemorrhage from an ectopic varix successfully treated with emergent glue embolization of mesenteric, omental, and periumbilical varices. Intraperitoneal bleeding from ectopic varices in cirrhosis patients is a rare, life-threatening condition. Consideration and recognition of ectopic variceal hemorrhage in patients with cirrhosis can facilitate prompt life-saving treatment in a population susceptible to significant morbidity and mortality.

## Introduction

Varices are abnormally dilated veins resulting from portal hypertension and are present in approximately 50% of patients with cirrhosis [1]. Ectopic varices occur beyond the gastroesophageal junction and have been described in a variety of locations including the omentum, duodenum, and vagina [2]. Bleeding from ectopic varices accounts for up to 5% of all cases of variceal bleeding and is associated with high mortality [3-4]. Furthermore, management of ectopic variceal bleeding can be challenging in the absence of any evidence-based guidelines.

Abdominal pain is a common complaint in patients with cirrhosis resulting in high rates of presentation to emergency departments (EDs) [5]. Etiologies of abdominal pain in patients with cirrhosis may be related (e.g. ascites, spontaneous bacterial peritonitis, portal vein thrombosis) or unrelated (e.g. peptic ulcer disease, cholecystitis, musculoskeletal pain) to liver disease [6-7]. Patients with cirrhosis are likely to be medically complex and have higher rates of psychiatric illness, making the evaluation of abdominal pain challenging [8-9].

We present the case of a patient with a delayed diagnosis of hemoperitoneum secondary to ectopic variceal bleeding after initially presenting with right upper quadrant abdominal pain who was ultimately managed with glue embolization of omental varices.

## Case presentation

A 54-year-old male with alcoholic cirrhosis presented to the ED with right upper quadrant abdominal pain. His past medical history was significant for alcoholic cirrhosis diagnosed one year prior to admission with grade 1 esophageal varices and mild ascites; coronary artery disease, with stent placement six years prior, and hypertension. The patient had ongoing alcohol use at the time of presentation. The patient denied any hematemesis, hematochezia, or melena.

On initial evaluation, the patient appeared well with a blood pressure of 160/88 mmHg, heart rate of 96 beats/minute, respiratory rate of 24 breaths/minute, and a normal temperature. His abdominal exam was benign: his abdomen was soft and non-tender on palpation. Laboratory results were notable for lactic acid elevation of 2.7 mmol/L (0.7-2.0), hemoglobin was 14.5 g/dL (11.7-15.7), total bilirubin was 0.9 mg/dL (0.2-1.2), aspartate aminotransferase (AST) was 40 U/L (2-40), alanine aminotransferase (ALT) was 28 U/L (8-45), alkaline phosphatase (ALP) was 213 U/L (50-136), serum creatinine was 0.9 mg/dL (0.52-1.04), and urinalysis was normal. A non-contrast abdominal CT scan demonstrated no acute findings. The patient’s presenting pain resolved spontaneously and he was subsequently discharged home from the ED.

One week later, the patient returned to the ED with similar abdominal pain and was admitted to the hospital. Blood pressure was 125/70 mmHg, heart rate was 89 beats/minute, respiratory rate was 18 breaths/minute, and the patient was afebrile. The abdominal exam showed mild distension on inspection but again was non-tender on palpation throughout. On laboratory evaluation, the patient’s hemoglobin was 13.3 g/dL, platelet count was 170 x 10^3^/uL (150-450 x 10^3^), and white blood cell count was 7 x 10^3^/uL (4-11 x 10^3^). Serum creatinine remained normal at 0.7 mg/dL. However, on this occasion, the patient had a direct hyperbilirubinemia with a total bilirubin of 12.1 mg/dL. AST, ALT, and ALP remained similar to prior. Magnetic resonance cholangiopancreatography was obtained and showed a normal biliary tract with no evidence of stricture. Abdominal paracentesis was not performed due to an inadequate amount of ascites. The patient’s pain again spontaneously resolved and after three days in the hospital, he was discharged with a diagnosis of acute decompensated cirrhosis of unclear etiology.

One day after discharge from the hospital, the patient re-presented to the ED with sudden onset of severe abdominal pain. On initial evaluation, the patient was hypotensive with a blood pressure of 87/60 mmHg, and tachycardic with a heart rate of 118 beats/minute. On general inspection, the patient was alert but pale and jaundiced. Abdominal examination was notable for mild distention with diffuse tenderness on palpation without rebound or guarding. The remainder of the physical examination was unremarkable.

At this time, the patient’s laboratory results revealed a white blood cell count of 14.2 x 10^3^/uL, hemoglobin of 10.8 g/dL, and lactic acid of 4.5 mmol/L. Serum creatinine had increased to 1.4 mg/dL and total bilirubin had slightly increased to 14.3 mg/dL. AST was now elevated at 124 U/L while ALT and ALP were stable at 58 and 159 U/L, respectively.

After fluid resuscitation, the patient became hemodynamically stable. Broad-spectrum antibiotics were administered at the same time due to a concern for sepsis from an intra-abdominal infection. Twelve hours later, the patient’s hemoglobin decreased to 7.0 g/dL, prompting the administration of two units of packed RBCs.

**Figure 1:**
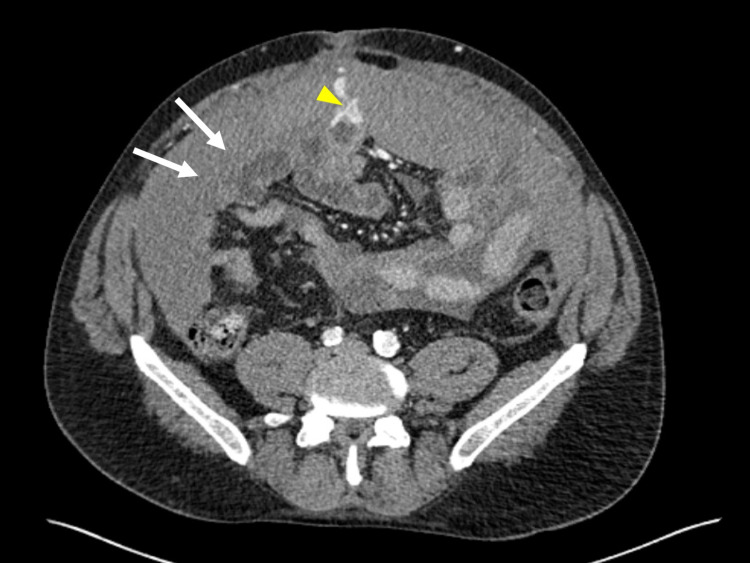
CT with angiography showing hemoperitoneum (white arrow) with possible source of hemorrhage from portosystemic collateral (yellow arrow) in the region of greater omentum and small bowel wall; axial view.

**Figure 2:**
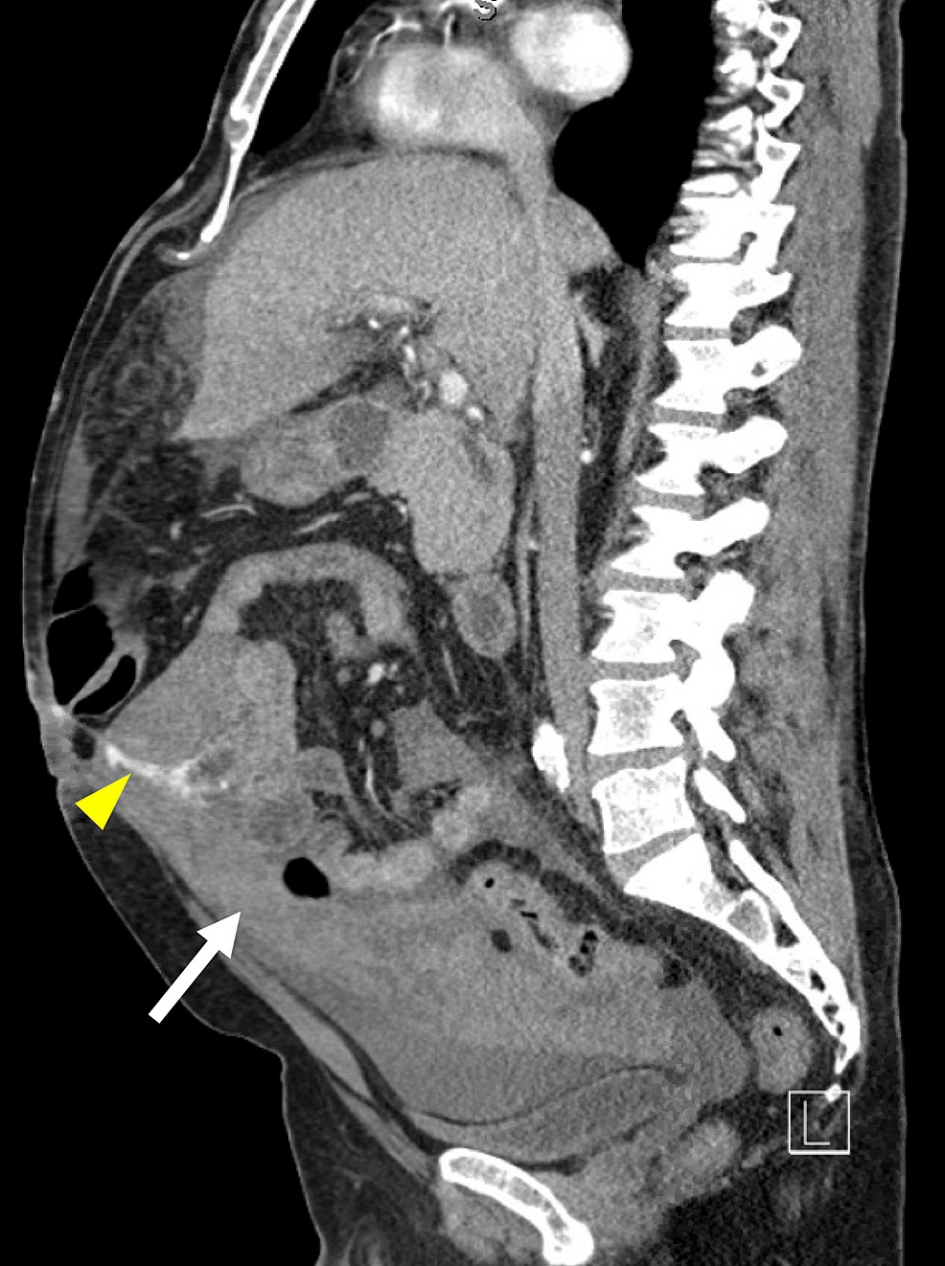
CT with angiography showing hemoperitoneum (white arrow) with possible source of hemorrhage from portosystemic collateral (yellow arrow) in the region of greater omentum and small bowel wall; sagittal view.

An emergency abdominal non-contrast CT showed mild to moderate hemoperitoneum. An immediate subsequent abdominal CT angiogram identified a potential source of hemorrhage at a portosystemic collateral in the region of the greater omentum and small bowel wall (Figures 1-2). No active extravasation was seen. CT also demonstrated cirrhosis with portal hypertension and portosystemic collateralization at splenorenal, periesophageal, and perigastric regions.

**Figure 3:**
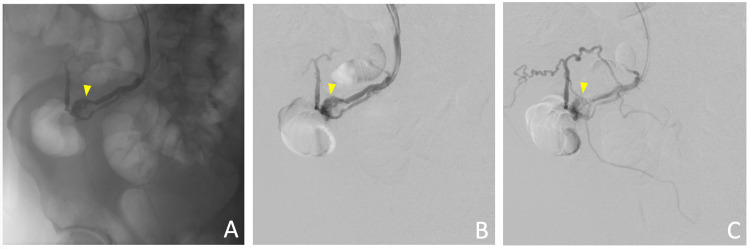
Portal venogram showing superior mesenteric venous flow into collateral vessels in the periumbilical region; (A) Scout film of the portal venogram; (B-C) Sequential venogram.

**Figure 4:**
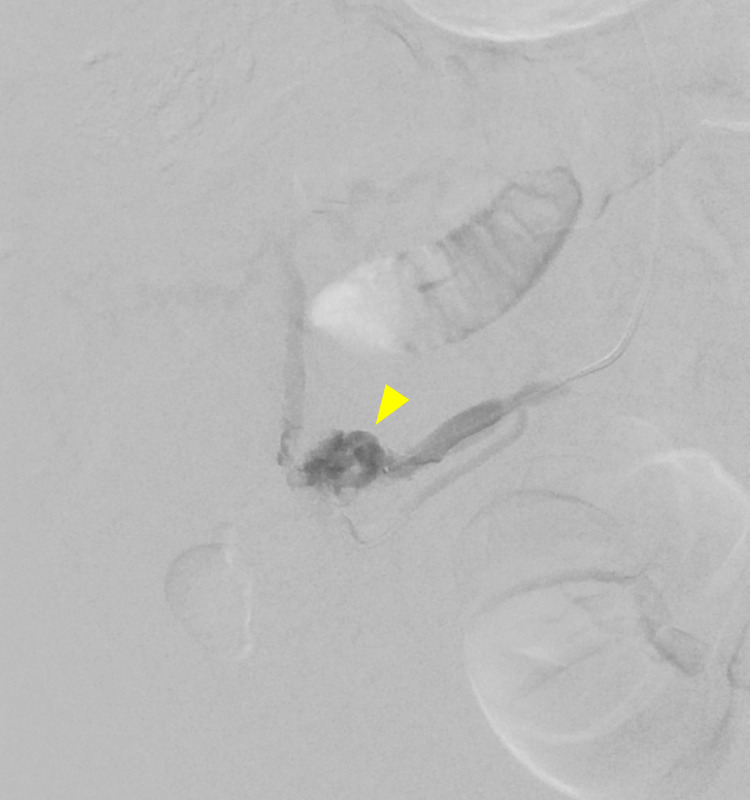
Venogram of glue embolization of branches of superior mesenteric vein, via microcatheter.

**Figure 5:**
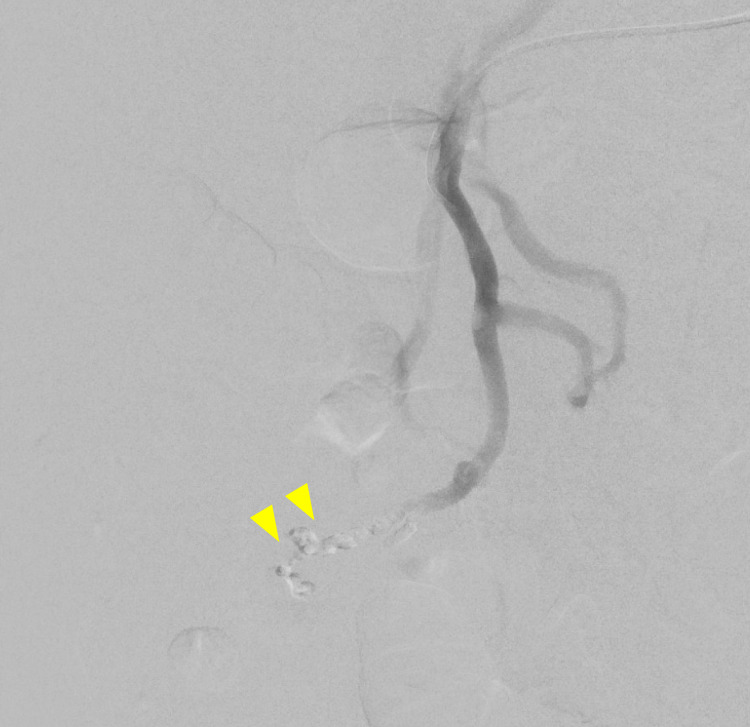
Venogram of post glue embolization of branches of superior mesenteric vein showing no persistent flow into the nidus.

Interventional radiology was consulted as the patient was still requiring blood transfusion. The patient underwent a portal venogram which demonstrated superior mesenteric venous flow into collateral vessels in the periumbilical region which fed a tangle of abnormal vessels likely representing the site of the recent hemorrhage (Figure 3). With the result from the venogram, the patient successfully underwent emergent glue embolization of mesenteric, omental, and periumbilical varices using an ultrasound-guided, trans-splenic approach (Figures 4-5). The splenic collateral vessels were also embolized with coils. There were no immediate procedural complications and the patient received five days of IV piperacillin-tazobactam while in hospital. The patient’s abdominal pain soon resolved and he was discharged on post-procedure day four.

One month following admission, upper endoscopy was performed and showed large esophageal varices, which were successfully treated with band ligation. Two and a half years after the event, the patient remains well with no recurrent bleeding episodes and his liver disease has remained well-compensated despite ongoing alcohol use at two to four drinks a day.

## Discussion

Abdominal varices are commonly found in patients with portal hypertension, most often related to chronic liver disease. Esophageal varices are the most common type of abdominal varices in patients with cirrhosis with an annual incidence of 3%-10%, while the prevalence is higher in patients with more advanced liver disease [10-11].

Ectopic varices are defined as dilated portosystemic collateral veins at sites that occur beyond the gastric or esophageal regions [11]. The pathogenesis of these varices is similarly related to increased pressure in portal circulation due to liver parenchymal disease, or obstruction of extrahepatic veins as seen with splenic vein or portal vein thrombosis [12]. Although ectopic varices are often observed on cross-sectional imaging in patients with cirrhosis and portal hypertension, bleeding is rare [13]. However, when rupture of these varices does occur, it is usually severe and has a higher risk of rebleeding [14].

In patients with cirrhosis and acute anemia without signs of overt gastrointestinal bleeding, it is important to consider possible hemorrhage from ectopic abdominal varices in the differential diagnosis. Although bleeding from ectopic varices is rare, it is potentially fatal and should be considered in at-risk patients. In our case, the diagnosis was delayed for 10 days as the patient did not have the classic initial presentation of acute anemia and hypotension expected in an acute hemorrhage. Furthermore, a CT abdomen without contrast was obtained at the initial ED visit, which has a low sensitivity for detection of bleeding in the absence of significant hemoperitoneum. In contrast, a CT angiogram of the abdomen would have been the preferred diagnostic modality as well as the most timely therapeutic intervention. CT angiogram is able to identify the bleeding site with active extravasation of contrast media [15-16].

There is no standard guideline for managing bleeding from ectopic varices due to low prevalence and a lack of randomized control trials [1, 11]. Initial management includes stabilization of hemodynamics with volume resuscitation and somatostatin analogs [1, 17-18]. If intraluminal bleeding occurs, hemostasis can be achieved with an endoscopic procedure. If bleeding is refractory to endoscopic therapy or occurs in the peritoneal cavity, like in our presenting case, transjugular intrahepatic portosystemic shunt (TIPS) or transvenous obliteration procedure will be needed to achieve hemostasis [1, 19]. Both interventions have similar rebleeding rates of 21%-37% for TIPS and 17%-31% for transvenous obliteration procedure when utilized for management of bleeding ectopic varices [19].

In a literature review of published cases, we identified eight previous case reports of hemoperitoneum in the setting of bleeding ectopic abdominal varices [3, 20-26]. Four patients underwent exploratory laparotomy with ligation with 100% mortality [21, 24-25]. One patient was initially diagnosed with bleeding from esophageal varices and underwent successful emergent TIPS placement. However, the patient later decompensated with massive bleeding and was later found to have gallbladder varices bleeding with hemorrhagic peritoneum on autopsy [23]. Another patient was found dead at home with the diagnosis of ectopic varix bleeding also made on autopsy [3].

Only two previous case reports involved an interventional radiology procedure [20, 22]. The first patient was a 37-year-old male with cirrhosis due to non-alcoholic fatty liver disease who received coil embolization of a varix emanating from the superior mesenteric vein [20]. He successfully underwent orthotropic liver transplant during the same hospitalization. The second patient was a 49-year-old male patient with alcoholic cirrhosis who received glue embolization of right colic and ileocolic varices [22]. Both patients survived without any recurrent bleeding during the follow-up period. Our patient underwent successful coil-assisted, glue embolization of bleeding mesenteric, omental, and periumbilical varices and has done well, over the subsequent follow-up period, similar to the other patients who underwent an interventional embolization procedure.

## Conclusions

In conclusion, we present a case of life-threatening bleeding from ectopic intra-abdominal varices presenting as abdominal pain and successfully treated with glue embolization. Ectopic varices are relatively common in patients with cirrhosis but have the potential to cause fatal bleeding. Consideration and recognition of this rare complication of portal hypertension are important in order to facilitate prompt treatment in a patient population already at high risk of significant morbidity and mortality.
